# The interaction of diet-induced obesity and chronic stress in a mouse model of menopause

**DOI:** 10.1016/j.yhbeh.2026.105947

**Published:** 2026-05-14

**Authors:** Nadja Knox, Ali Yasrebi, Daniel Caramico, Katherine A. Denney, Kimberly Wiersielis, Benjamin A. Samuels, Troy A. Roepke

**Affiliations:** a Department of Animal Sciences, School of Environmental and Biological Sciences, Rutgers University, New Brunswick, NJ, 08901, United States of America; b Graduate Program in Endocrinology and Animal Biosciences, Rutgers University, New Brunswick, NJ, 08901, United States of America; c Department of Psychology, School of Arts and Sciences, Rutgers University, Piscataway, NJ, 08854, United States of America

**Keywords:** Menopause, Diet-induced obesity, Chronic stress, Behavior, Bed nucleus of the stria terminalis

## Abstract

Menopause is characterized by the cessation of ovarian hormone production. During postmenopause, cisgender women face increased risks of obesity, cognitive decline, and mood disorder. Mood disorders are associated with exposure to chronic stress. We investigated the combined effects of a high-fat diet (HFD) and chronic stress exposure in a mouse model of menopause using 4-vinylcyclohexene diepoxide (VCD), a selective ovotoxicant that gradually depletes ovarian follicles and hormones. Starting at 6 months, 82 female WT C57BL/6 J mice received sesame oil or VCD (130 mg/kg i.p.) 5 days per week for 3 weeks. One month after injection, mice were fed either low-fat diet (LFD) or HFD for 8 weeks followed by 6 weeks of chronic variable mild stress (CVMS). Post-CVMS, mice were either processed for gene expression of the anterodorsal bed nucleus of the stria terminalis (adBNST) or behavior tests to assess cognitive and anxiety-related behaviors. Plasma samples were collected to analyze metabolic hormones and corticosterone levels. VCD-treated HFD-fed mice had higher fat and body mass, and elevated fasting glucose levels compared to controls and more pronounced avoidance behaviors and cognitive impairments. VCD-treated LFD-fed mice exhibited less exploration of novel objects and open spaces compared to sesame oil and HFD counterparts. VCD elevated corticosterone levels on LFD and increased BNST *Adcyap1* (PACAP) gene expression on HFD. These findings highlight cognitive repercussions of estrogen deficiency and suggest a potential protective effect of a HFD against some of the adverse outcomes associated with menopause. Our study emphasizes the importance of considering dietary and hormonal interactions in the development of therapeutic strategies.

## 1. Introduction

Menopause is a naturally occurring physiological process occurring in humans, higher primates, and whales (Sievert, 2025). Typically, in humans, menopause is confirmed by twelve months without a menstrual cycle due to the depletion of follicles, a decrease in their sensitivity to gonadotropins, and the cessation of ovarian hormone production. During the perimenopausal period, steroid production fluctuates due to shifts in the menstrual cycle and the lack of ovulation leading to irregular menses. The drop in estradiol has been shown to affect neurotransmitter systems and brain regions involved in mood regulation, leading to heightened susceptibility to mood disorders during and after the menopausal transition (Reis et al., 2014). Furthermore, progesterone production decreases and releases the negative feedback onto the hypothalamus and pituitary leading to elevated follicle stimulating hormone (FSH) and luteinizing hormone (LH). As progesterone is also neuroprotective, the loss of progesterone may also contribute to mood and cognitive decline while the increase in FSH or LH may facilitate these neurological conditions (Valera et al., 2025), These changes increase the risk in cisgender women of developing mental health disorders such as anxiety, depression and cognitive decline (Dalal and Agarwal, 2015; Li et al., 2016; Bauld and Brown, 2009; Freeman et al., 2007; Reis et al., 2014).

Obesity, prevalent in a substantial portion of the female population worldwide, further exacerbates mental health risks, including anxiety and depression, and cognitive decline (Abdalla et al., 2022; James et al., 2026). The relationship between obesity and mental health is multi-faceted, involving both physiological and psychosocial factors (Patterson et al., 2013; Spencer et al., 2017; Tomiyama et al., 2018). Excess adipose tissue can lead to chronic inflammation and altered endocrine function, which are implicated in the development of mood disorders (Rosenbaum et al., 2005; Spencer et al., 2017). Depletion of 17β-estradiol in both mice and humans is associated with greater adiposity whether through surgical procedures, chemical exposures, or biological processes (Sui et al., 2023; Mauvais-Jarvis et al., 2013). Obesity and stress are intricately connected, with each exacerbating the effects of the other (Tomiyama et al., 2018). Additionally, the stigma associated with obesity and the resultant psychological stress contribute significantly to the elevated risk of mental health issues (Puhl and Latner, 2007; Tomiyama et al., 2018).

Stress disrupts cognitive functions, including self-regulation, which can contribute to unhealthy behaviors such as excessive intake of high-calorie foods, reduced physical activity, and poor sleep quality (Tomiyama et al., 2018). Recently, a chronic variable stress paradigm was reported to induce obesity in ~20% of male mice on a normal chow diet in part by hyperactivating the hypothalamic-pituitary-adrenal (HPA) axis in male mice but not female mice (Liu et al., 2024). Chronic stress is also a major risk factor for mental health disorders, including anxiety and depression. The persistent activation of the stress response system leads to dysregulation of the HPA axis, resulting in prolonged exposure to stress hormones like cortisol. This physiological stress response impacts brain function and structure, particularly in regions involved in emotional regulation, such as the hippocampus and amygdala (Kang et al., 2023). Chronic stress also exacerbates preexisting mental health conditions and can contribute to the onset of new disorders.

Corticotropin-releasing hormone (CRH) is a key regulator of the HPA axis, the central stress response system. CRH and its receptors are found in various brain regions, including the bed nucleus of the stria terminalis (BNST) (Peng et al., 2017). CRH is released in response to stress and triggers the release of adrenocorticotropic hormone (ACTH) from the pituitary gland, which in turn stimulates the production of stress hormones from the adrenal glands. CRH and other stress-related factors have been implicated in the regulation of both cognition and feeding behavior, with elevated CRH levels being associated with anxiety, depression, and changes in appetite (Hu et al., 2020). Chronic variable mild stress (CVMS) and acute activation of the oval bed nucleus of the stria terminalis (ovBNST) lead to maladaptive behaviors in rodents, showing that chronic mild stress activates CRH signaling and CRH neurons in the ovBNST in male mice (Hu et al., 2020). We also reported that CVMS augmented CRH signaling, increased miniature excitatory post-synaptic activity, and reduced M-current activity within the oval nucleus of the BNST in male mice (Hu et al., 2020). Another recent study from our lab demonstrated that CVMS induced sex-associated differences in avoidance behavior in mice with increasing avoidance in stressed proestrous females (Degroat et al., 2024). A subsequent study found that CVMS reduced the M-current in BNST CRH neurons from male mice but not from intact (diestrus) female mice while differentially regulating 217 genes in males and only 1 gene in females in the anterodorsal (ad) BNST (Degroat et al., 2025).

Previous studies have found that CRH and associated genes (CRH receptors 1 and 2 (CRHR1 and CRHR2)), pituitary adenylate cyclase-activating polypeptide (PACAP), PACAP receptor (PAC1r), and striatal-enriched tyrosine phosphatase (STEP), are involved in the response to chronic stress (Hu et al., 2020; Maita et al., 2022; Lepeak et al., 2023). CRHR1 and CRHR2 are the receptors through which CRH exerts its effects, with CRHR1 primarily mediating stress-related responses and CRHR2 involved in modulating the stress recovery process (Baritaki et al., 2019). PACAP and its receptor PAC1r are involved in neuroplasticity and stress responses, influencing both neural and hormonal pathways (Stroth et al., 2011). STEP has been shown to have a role in regulating the strength of synaptic connections and, consequently, learning and memory processes (Xu et al., 2012; Reinhart et al., 2014).

Therefore, we investigated the combined effects of a high-fat diet (HFD) and chronic stress using a mouse model of menopause: 4-vinylcyclohexene diepoxide (VCD) treatment. VCD, a selective ovotoxicant thatgradually depletes ovarian follicles and hormones, mimicking the natural menopausal transition. Unlike ovariectomy, which causes abrupt hormonal loss, VCD provides a more accurate representation of age-related hormonal changes, a perimenopausal transition, ovary-intact estrogen deficiency due to follicular depletion while avoiding the stress of surgery. Previous studies have confirmed the ability to induce this level of reproductive senescence from VCD exposure (Van Kempen et al., 2014; Sui et al., 2023). This experiment builds on our previous VCD studies (Sui et al., 2023), by investigating how menopause and HFD feeding (or diet-induced obesity (DIO)) influence the behavioral response to a chronic stressor in avoidance and cognition/memory, and the transcriptome of the adBNST. That previous study only characterized similar cognitive behavioral outcomes in VCD- treated females, without stress exposure, finding impaired spatial memory in VCD-treated females on a HFD (Sui et al., 2023). Thus, the current approach aims to address gaps in understanding how these factors (diet, VCD, stress) interact and impact physiological and behavioral outcomes, an area not thoroughly explored to date. While our experiment does not have a no-stress comparison, the information gathered will increase our understanding of the interactive impact of diet and menopause on behavioral outcomes under stressful conditions that may increase the risk for mood disorders and cognitive impairment. We predict that in response to a stressor, VCD-treated mice will exhibit greater avoidance behaviors and impaired memory compared to oil-treated (control mice) and that HFD may ameliorate some of these impairments. This study will provide valuable insights into the mechanisms underlying postmenopausal health risks and emphasizes the importance of considering dietary and hormonal interactions in the development of therapeutic strategies.

## 2. Methods and materials

### 2.1. Animal care and 4-vinylcyclohexene diepoxide (VCD) administration

**Fig. 1.**
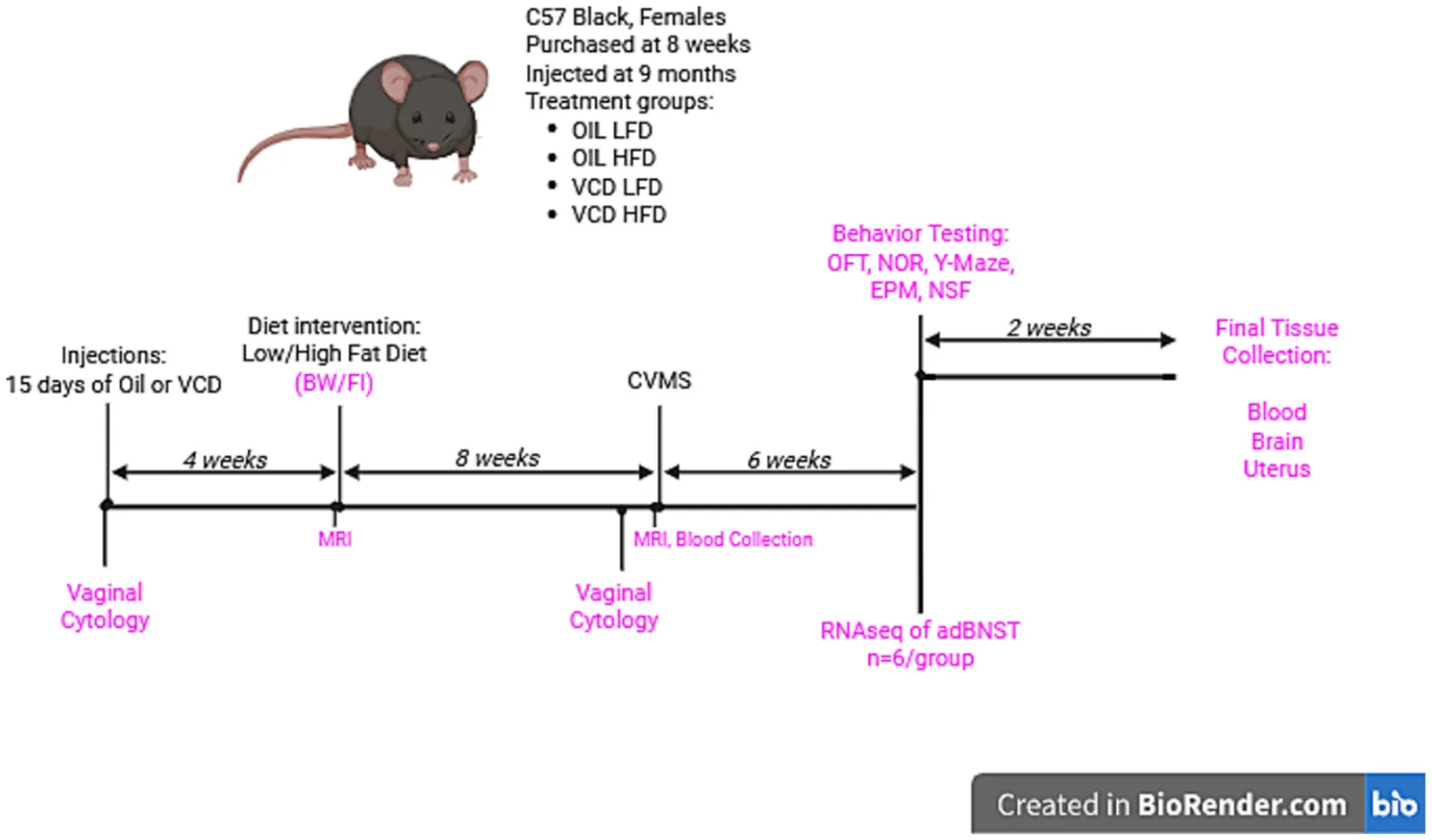
Schematic of experimental timeline. Study mice were divided into four treatment groups: OIL LFD, OIL HFD, VCD LFD, and VCD HFD. All mice were injected intraperitoneally (either oil or 130 mg/kg VCD) 5 days per week for 3 weeks, starting at 9 months. Vaginal cytology and MRI were performed before and after 8 weeks of diet introduction and body weight, food intake monitoring. Chronic variable mild stress (CVMS) paradigm was conducted for a six-week period. RNA sequencing as well as qPCR of the anterodorsal bed nucleus of the stria terminalis (adBNST) was conducted post-CVMS to analyze gene expression changes on a subset of mice. Behavioral testing (Novel Object Recognition, Y-Maze, Elevated Plus Maze, Novelty Suppressed Feeding), were performed following stress exposure on another subset.

Procedures adhered to the standards set by the National Institutes of Health and were authorized by the Rutgers Institutional Animal Care and Use Committee. Adult female C57BL/6 J mice, purchased from The Jackson Laboratory (Jackson Laboratory, Bar Harbor, ME) at 8 weeks of age, were exclusively used in our study. All mice were group housed and fed a standard chow (Lab Diet 5 V75) prior to introduction of their matched low- or high-fat diets. Apart from when mice were actively being exposed to stressors, these mice were housed in a controlled environment with a temperature of 22 °C and subjected to a 12-h light/dark cycle, with ad libitum access to food and water. After 6 months in our facility, mice were pair housed and administered either sesame oil (OIL) or 4-vinylcyclohexene diepoxide (VCD) via intraperitoneal injections at a dose of 130 mg/kg 5 days per week for 3 weeks (Fig. 1). Animals were given 4 weeks to recover from their injections prior to diet intervention. A subset of animals was euthanized at 18 weeks post injection for RNA sequencing analysis (*n* = 6/group) or at 19–20 weeks post behavior testing observations (*n* = 11–16/group) for qPCR gene expression.

### 2.2. Vaginal cytology

Vaginal cytology (VC) of mice was performed using a lavage technique followed by analysis of the collected cells on a slide. This method involved gently flushing the vaginal canal with water, which was then transferred onto a glass slide and spread evenly to create a monolayer. The slide was examined under a light microscope to identify and classify the different stages of the estrous cycle (Caligioni, 2009; Cora et al., 2015). The first round of VC was performed for two weeks immediately prior to injections. The second round of VC was performed for 14 uninterrupted days during the 6th through 8th week of diet intervention, starting 10 weeks post injection. Ovarian failure was confirmed when VCD animals were observed to be in stages of low ovarian hormone state (either metestrus or diestrus) for significantly more days (*p* = 0.024, data not shown) than their oil counterparts.

### 2.3. Diets and metabolic phenotyping

Mice were randomly assigned matched diets four weeks after their last round of injections; they remained on their respective matched diets through the duration of the study. Diet and food intake analysis involved feeding the mice either a Low-Fat Diet (LFD 10% kCal fat, Cat D12450K; Research Diets, New Brunswick, NJ) or a High-Fat Diet (HFD 45% kCal fat, Cat D12451; Research Diets, New Brunswick, NJ) ad libitum throughout the experiment. Food intake was monitored weekly for eight weeks prior to stress paradigm and behavior testing. The body composition of the mice was assessed using an EchoMRI 3-in-1 Body Composition Analyzer (Echo Medical Systems, Houston, TX, USA) both before and after the dietary intervention. This non-invasive imaging technique collected body composition parameters such as fat mass and lean mass. Additionally, mice were fasted for 5 h, and baseline fasted glucose levels were determined prior to diet intervention.

### 2.4. Chronic variable mild stress (CVMS)

The chronic variable mild stress (CVMS) paradigm, previously used in published work by our lab (Degroat et al., 2025), involved subjecting mice to up to two daily stressors over a six-week period. These stressors included alterations in the light/dark cycle (such as overnight illumination), temperature fluctuations (such as 15 min of 4 °C exposure), bedding modifications (including removal of bedding, substitution with wet bedding or one centimeter of room-temperature water, or introduction to the bedding of a novel mouse of the same sex), frequent cage changes (5 or 6 times per day), forced swimming (either in 21 °C water for 4 min or 4 °C water for 2 min), isolation stress lasting for 8 h or overnight, restraint stress for 1 h, cage tilting at a 45° angle for 8 h, and exposure to predator sounds for 15 min. Stressors were randomized throughout the 6-week period.

### 2.5. Behavior testing

Behavior tests took place in the order of open field test, novel object recognition, Y-maze, elevated plus maze, and finally novelty suppressed feeding. All tests were done in succession, allowing mice to remain undisturbed in their home cages for ~22–24 h between tests. Mice were transferred from their home room into a behavior testing room and allowed 24 h of habitation to the behavior room prior to testing. All tests were started approximately 2 h after lights on. Visual cues were placed on the walls and were not moved throughout all the tests. An empty opaque cage was used as a holding cage to keep mice away from visual cues during inter-trial delay. The interior area of testing arenas and the holding cage were sanitized by MB10 during each inter-trial-delay as well as between each testing trial to minimize olfactory cues. All tests were recorded in real-time, and videos were analyzed via Any-Maze software (ANY-maze, Version 6, Stoelting, USA).

### 2.6. Open field test and novel object recognition

The Novel Object Recognition (NOR) test aims to evaluate the ability of mice to recognize and remember novel objects, providing insights into their recognition memory abilities. Mice had five consecutive days of habituation during which they were allowed to explore the empty open field for five minutes. The first habituation day of the NOR is effectively a 5-min Open Field Test (OFT). OFT is a behavioral test used to assess anxiety-like behavior and general locomotor activity, where a mouse is placed in a large, open arena, and allowed to explore freely. The 40×40 cm OFT field is partitioned into zones of interest and analyzed by section - corners (outer 5 cm sections of either quadrant), outer perimeter (outer 10 cm), 20×20 cm middle (20 cm center), and 10×10 cm middle (10 cm center). More time spent in the corners or perimeter or less time in the center zones suggests an increase in avoidance behavior.

On the sixth day, each mouse experienced a five-minute exposure trial where two new identical objects (either two 250-ml glass flasks or two iron brackets) were again presented in two of the four quadrants of the open field, followed by a five-minute inter-trial delay. One of the familiar objects was replaced, in a counter balanced fashion, with a novel object while the second familiar item stayed in the same location as it had been during the exposure trial. The mouse was reintroduced into the open field and recorded exploring for 5 min as the test trial. Various locomotor and orientation measurements pertaining to each object were recorded and analyzed. More time orientated towards or spent with the novel object indicates better hippocampal-independent memory.

### 2.7. Y-maze

Y-maze test assesses spatial working memory and exploratory behavior by measuring behavior in mice navigating through the maze’s three arms (each arm being 30 cm in length). One arm was designated the starting arm, and the other two arms were defined as either the known or novel arm for each test which was counterbalanced between mice. For the exposure trial, the mouse was placed at the distal end of the starting arm, facing towards the center of the maze and allowed to explore both the starting arm and known arm for five minutes undisturbed. The mouse was then placed in a holding cage for a five-minute inter-trial delay, during which the field divider was removed from the novel arm and the mouse was returned to the Y-maze to freely explore all three arms for five additional minutes. Locomotion, time and location relative to each of the three arms (starting arm, known arm, unknown arm) during the final 5-min exploration window were recorded and analyzed. Less time spent in the novel arm suggests an impaired spatial or hippocampal-dependent memory.

### 2.8. Elevated plus maze

The Elevated Plus Maze (EPM) test assesses avoidance behavior in mice. Mice were introduced into the central area of a plus-shaped maze (each arm being 28 cm in length), positioned facing an open arm, and allowed to explore for a duration of 5 min. Parameters associated with movement and location relative to either the closed or open (with an emphasis on open arm ends- the most distal point of the open arm) were analyzed. More time spent in the closed arm or less time in the open arms indicates greater avoidance behaviors.

### 2.9. Novelty suppressed feeding

The Novelty Suppressed Feeding (NSF) test helps assess avoidance behavior producing a conflict between the desire to eat after fasting and the fear of venturing into a brightly lit novel arena. Mice were subjected to a 24-h food deprivation period, starting the day before testing, and were subsequently placed in holding cages at 8:00 A.M. on the testing day. A longer latency to eat in the novel arena indicates greater avoidance or a reduced motivation to eat.

### 2.10. Blood and tissue collection

“Pre-stressed” mice were briefly sedated under 2% isoflurane and blood was collected via submandibular bleeds as serum. Post-experimentation, animals were euthanized via decapitation following sedation with ketamine (100 μL of 100 mg/ml) after a one-hour fast. Brain, liver, ovaries, and gonadal white adipose tissue (GWAT) were harvested and stored at −80 °C for future analysis. Trunk blood was collected in K2-EDTA tubes and processed for plasma (Mamounis et al., 2018). Brain slices were then transferred to RNAlater (Life Technologies, Inc., Grand Island, NE, USA) and stored overnight at 4 °C. The BNST was dissected from slices using a dissecting microscope and stored at −80 °C for RNA sequencing analysis 1 h after the last CVMS stressor (wet bedding) as previously described (Degroat et al., 2024) or for qPCR one week after the last behavioral assay (Novel Suppressed Feeding).

### 2.11. Hormone assays

Plasma analysis of metabolic hormones was conducted using the Luminex^®^ MagPix^™^ multiplex system (Millipore). Plasma samples were prepared by adding 4-(2-aminoethyl) benzenesulfonyl fluoride hydro-chloride (ABESF, 1 mg/ml) to each collection tube. The prepared plasma samples were used for multiplex analysis using Millipore plate #MMHE-44 K, for leptin, ghrelin, and insulin. Pre- and post-stress serum or plasma samples were analyzed for corticosterone (CORT) concentrations via ELISA (RTC002R; Biovendor; Degroat et al., 2024).

### 2.12. RNA sequencing

RNA samples extracted from the BNST were subjected to RNA-sequencing. Mice (*n* = 6/group) were euthanized after a one-hour humid bedding stressor, and brain slices were extracted and stored in RNAlater solution and later microdissected to isolate adBNST tissue, which was preserved in RNAlater and stored at −80 °C until RNA isolation. RNA extraction was performed utilizing the Ambion RNAqueous Micro Isolation kit (Thermofisher), and RNA integrity was assessed using an Agilent 2100 Bioanalyzer with an RNA 6000 Nano kit. All samples had an RNA Integrity Number of 8 or higher. The RNA samples were then sent to the JP Sulzberger Columbia Genome Center (New York, NY) for sequencing. Library preparation was carried out using the TruSeq Stranded mRNA Library Prep Kit (Aviti Element), and the pooled libraries were sequenced on the Illumina NovaSeq 6000 platform with 100 bp paired-end reads, aiming for a sequencing depth of 40 million reads. Subsequently, the resulting reads underwent quality trimming using the FastX Toolkit (http://hannonlab.cshl.edu/fastx_toolkit/) with a minimum quality score of 20.0. Alignment to the mm10 reference genome was performed using STAR along with the NCBI RefSeq mm10 gene annotation, and reads were counted using FeatureCounts (Dobin et al., 2013; Liao et al., 2014). The data were subsequently imported into RStudio (version 2023.12.0 + 369), to conduct differential expression analysis using DESeq2 with the design formula: design = ~ diet + treatment (oil vs. VCD) + the interaction of diet and treatment (which is written as diet:treatment within the R code) (Love et al., 2014). Principal component analysis (PCA) was performed using RStudio to identify any outliers. Raw sequencing data can be accessed at GEO#: GSE324900.

### 2.13. Quantitative real-time PCR

BNST samples collected for qPCR were processed as above for RNA extraction. cDNA was synthesized from total RNA using Superscript III reverse transcriptase (Life Technologies, Inc), 4 μL 5× buffer, 25 mM MgCl_2_, 10 mM deoxynucleotide triphosphate (Bio-Rad Laboratories, Inc), 100 ng random hexamer primers (Bio-Rad Laboratories, Inc), 40 U/μL RNasin (Bio-Rad Laboratories, Inc), and 100 mM dithiothreitol in diethylpyrocarbonate-treated water in a total volume of 20 μL. Reverse transcription was conducted using the following protocol: 5 min at 25 °C, 60 min at 50 °C, and 15 min at 70 °C. The cDNA was diluted to 1:20 with nuclease-free water resulting in a final cDNA concentration of 0.5 ng/μL and stored at −20 °C. The adBNST tissue RNA was used for positive and negative controls (no reverse transcriptase) and processed with the experimental samples. Primers were designed and synthesized by Life Technologies using Clone Manager 5 software. Gene expression was assayed for *Crh* (F: AGGAGGCATCCTGAGAGAAGT; R: CATGTTAGGGGCGCTCTC), *Crhr1* (F: CGCAAGTGGATGTTCGTCT; R: GGCCCTGGTAGATGTAGT); *Crhr2* (F: AAGCTGGTGATTTGGTGGAC; R: GGTGGATGCTCGTAACTTCG); *Adcyap1* (PACAP, F: CAGAAGGCCAGTCACCTCTGTC; R: GAGAGTAGGCGAGCAGCCAAAG); *Adcyap1r1* (PAC1r, F: AACGACCTGATGGGACTAAAC; R: CGGAAGCGGCACAAGATGACC); and *Ptpn5* (STEP, F: GCACTGTGGCCGACTTTTG; R: CTGCACGGTGATCTCCACG). For qPCR, 4 μL of cDNA template was amplified using SSO Advanced SYBR Green (Bio-Rad Laboratories, Inc). The amplification protocol followed for all the genes: denaturing at 95 °C for 3 min followed by 40 cycles of amplification at 94 °C for 10 s (denaturing), 60 °C for 45 s (annealing), and completed with a dissociation step for melting point analysis with 60 cycles of 95 °C for 10 s, 65 °C–95 °C (in increments of 0.5 °C) for 5 s and 95 °C for 5 s. The reference genes used were *Gapdh* (F: TGACGTGCCGCCTGGAGAAA; R: AGTGTAGCCCAAGATGCCCTTCAG) and *Hprt* (F: GCTTGCTGGTGA AAAGGACCTCTCGAAG; R: CCCTGAAGTACTCATTATAGTCAAGGGC AT).

### 2.14. Statistical analysis

All parameters, unless otherwise specified, were analyzed using Prism (GraphPad Prism) with a significance threshold set at a *p*-value or adjusted p-value of <0.05. Prior to analysis, behavioral data was screened for outliers using the Grubbs’ test, employing a critical value of 0.05 as the cutoff. Subsequently, data were subjected to a two-way ANOVA (treatment, diet) followed by Fisher’s LSD *post-hoc* comparison, as we were only interested in comparison within treatment groups (diet or VCD treatments).

## 3. Results

### 3.1. Metabolic parameters

**Fig. 2.**
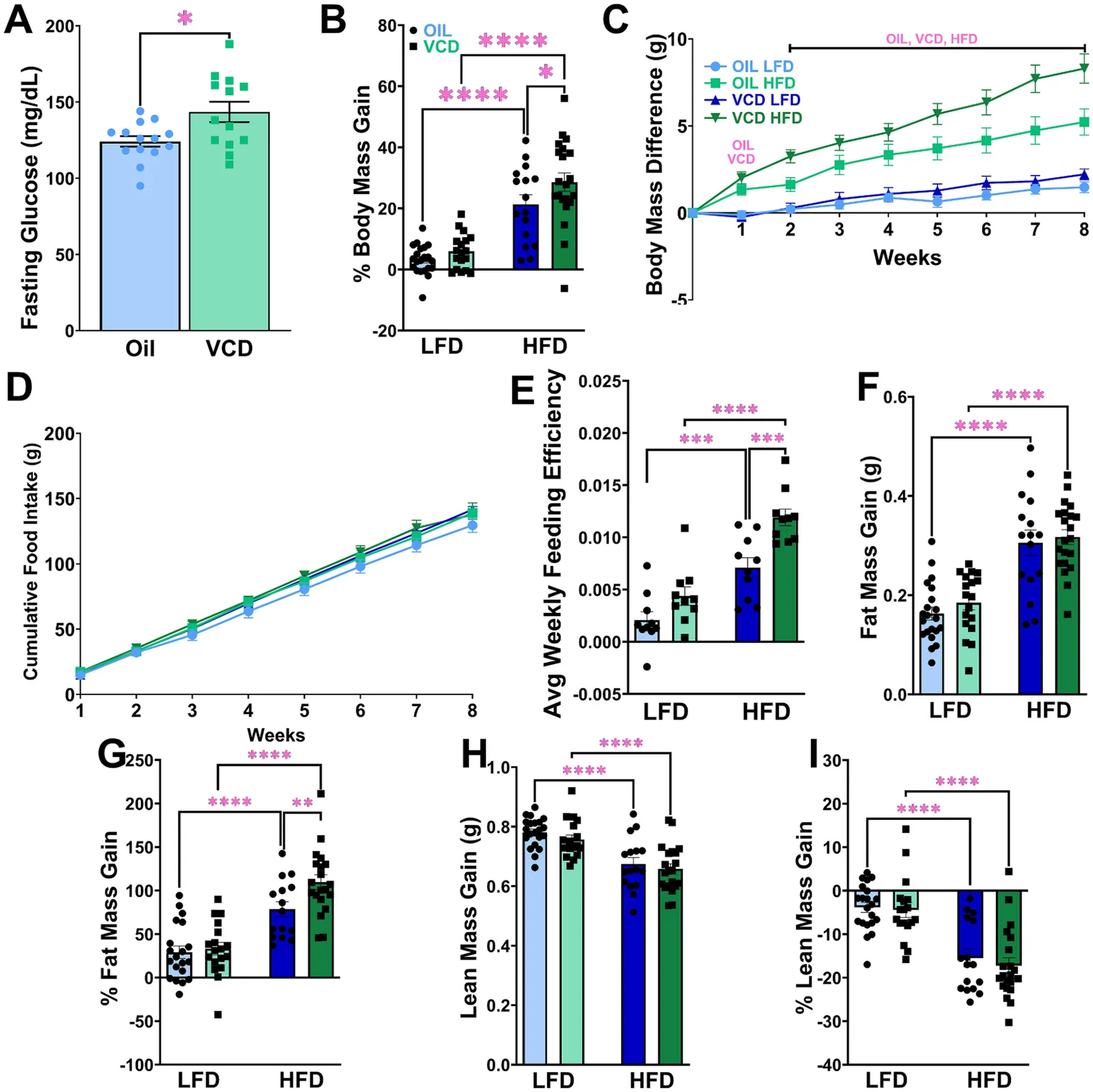
Body composition. Body composition results represent data collected both prior and post 8 weeks of matched diet introduction. (A) Blood glucose readings after a 5-h fast exposure prior to introduction of either LFD or HFD diets (B) Percent body mass gain (C) Weekly body mass difference (D) Grams of cumulative food intake (E) Average Feed efficiency, recorded as grams per kcal ingested, per box. (F) Grams of fat mass gain normalized to body mass (G) Percent fat mass gain (H) Grams of lean mass normalized to body mass (I) Percent lean mass gain. Data presented as mean +/−SEM and analyzed by two-way ANOVA with uncorrected Fisher’s LSD post-hoc comparisons (*, *P* = 0.01 to 0.05; **, *P* = 0.001 to 0.01; ***, *P* = 0.0001 to 0.001; ****, *P* < 0.0001).

As metabolic disturbances such as DIO may interact with menopause to influence the response to chronic stress (Patterson et al., 2013; Kumari et al., 2024; Shetty et al., 2024), we first characterized metabolic parameters in all groups. The unpaired *t*-test revealed a difference in five hour fasting glucose levels, prior to diet intervention, between the VCD and OIL groups (*t* = 2.624, df = 25, *p* = 0.0146), with the VCD group having a higher mean value (143.5 mg/dL) compared to the OIL group (124.1 mg/dL; Fig. 2A). HFD-fed, VCD-treated mice displayed a significant increase in percent body mass gained when compared to LFD-fed counterparts (*p* < 0.0001), as did HFD-fed oil-treated animals when compared to LFD-fed (p < 0.0001). Additionally, within the HFD groups, VCD-treated mice gain more body mass than oil-treated (*p* = 0.0307; Fig. 2B). There were also main effects of diet (F(8,664) = 35.38, *p* < 0.0001, *d* = 0.30), VCD (F(3,664) = 147.5, p < 0.0001, *d* = 0.46), and an interaction effect (F(24,664) = 4.204, *p* < 0.0001, *d* = 0.08) in cumulative gain in body mass (Fig. 2C). Both oil- and VCD-treated animals separated between diets (*p* < 0.05) starting from the first week continuing through the end of the 8 weeks of monitoring. Additionally, the same separation (p < 0.05) was seen between HFD-fed groups (oil vs. VCD) starting from the second week forward.

Analysis of cumulative food intake per animal revealed a treatment effect (F(3,275) = 7.767, *p* < 0.0001, *d* = 0.01; Fig. 2D) with LFD-fed, oil-treated females consuming less food overall despite no pairwise differences. Feeding efficiency is an indirect measure of metabolism with higher feeding efficiencies indicating lower metabolic costs and expenditure. Examining average weekly feeding efficiency per box revealed both diet (F(1,36) = 53.05, p < 0.0001, *d* = 0.49), and treatment effects (F(1,36) = 17.06, *p* = 0.0002, *d* = 0.16). Feeding efficiency was elevated by VCD-treatment (*p* = 0.0003) in HFD-fed females and lower in LFD-fed groups (OIL (p = 0.0002) and VCD (p < 0.0001)) compared to HFD-fed groups (Fig. 2E).

HFD-fed, oil- and VCD-treated mice exhibited higher fat mass (fat mass normalized to body mass) than their LFD counterparts (p < 0.0001 respectively; Fig. 2F), further affirming the observed diet effects (F (1,72) = 65.57, p < 0.0001, *d* = 0.47). HFD-fed, VCD-treated mice had higher percent fat mass gained compared to both HFD-oil (*p* < 0.01) and LFD-VCD (p < 0.0001). HFD-fed, oil-treated mice also had higher percent fat mass gained compared to their LFD-oil counterparts (p < 0.0001; Fig. 2G). Thus, percent fat mass had both diet (F(1,69) = 62.58, p < 0.0001, *d* = 0.45) and treatment (F(1,69) = 4.920, *p* = 0.0298, *d* = 0.04) effects. Both LFD-fed groups had higher normalized lean mass gains when compared to their HFD fed-counterparts (p < 0.0001; Fig. 2H). HFD also suppressed the percent lean mass gained in both treatment groups (p < 0.0001; Fig. 2I). Both normalized lean mass gained (F(1,72) = 39.73, p < 0.0001, *d* = 0.35), and percent lean mass gained (F(1,72) = 53.19, p < 0.0001, *d* = 0.43) were affected by diet. These data suggest that the depletion of follicles by VCD potentiates the effects of HFD feeding or DIO by increasing adiposity, increasing feeding, and reducing metabolism, presumably due to the decreases in 17β-estradiol production.

### 3.2. Behavioral analysis

**Fig. 3.**
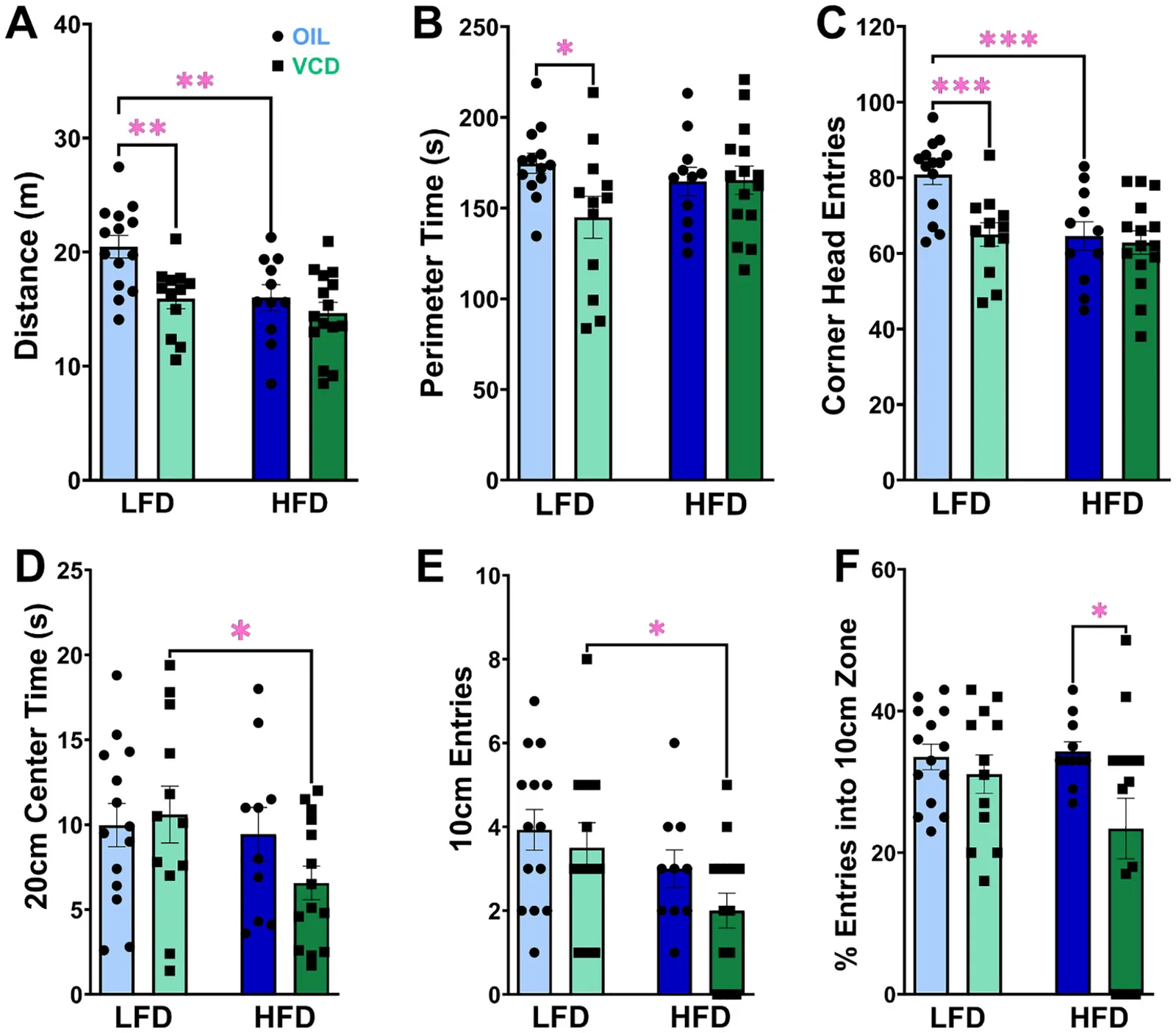
Open field test. Locomotor activity and avoidance behavior from the Open field test in oil- and VCD-treated females fed either LFD or HFD. (A) Total distance (meters) traveled (B) Total time (seconds) spent in perimeter (C) Number of head entries into corners (D) Total time (seconds) spent in 20 cm center (E) Total number of entries into the 10 cm center (F) Percent entries into the 10 cm zone. HFD-fed, VCD-treated mice exhibiting increased anxiety-like behavior compared to LFD-fed, oil-treated. Data presented as mean +/−SEM and analyzed by two-way ANOVA with uncorrected Fisher’s LSD post-hoc comparisons (*, P = 0.01 to 0.05; **, P = 0.001 to 0.01; ***, P = 0.0001 to 0.001).

The Open field test (OFT) field was analyzed by four parameters during analysis - the outer perimeter, corners, the inner 20 cm center, and the inner 10 cm center. Distance traveled was affected by diet (F (1,48) = 8.271; *p* = 0.0060, *d* = 0.12) and treatment (F(1,48) = 8.851; *p* = 0.0046, *d* = 0.13) with a decrease in distance traveled in LFD-fed, VCD-treated (*p* = 0.0021) and in HFD-fed, oil-treated (*p* = 0.003) groups (Fig. 3A). Perimeter time decreased in LFD-fed, VCD-treated females compared to oil-treated (*p* = 0.0165) and a trending effect observed between VCD-treated females (*p* = 0.0837, Fig. 3B). Interestingly, the number of entries made into the perimeter were affected by diet (F(1,47) = 5.487; *p* = 0.0234), *d* = 0.09) and there were differences within oil-treated mice (*p* = 0.0291). The corners are considered the most protective or safest space of the OFT. Corner head entries were affected by diet (F(1,48) = 8.482; *p* = 0.0054, *d* = 0.12), treatment (F (1,48) = 7.667; *p* = 0.0080, *d* = 0.11) and an interaction (F(1,48) = 5.012; p = 0.0298, *d* = 0.07). Between LFD-fed females, VCD treatment reduced the number of entries (*p* = 0.0009) and HFD reduced entries between oil-treated groups (*p* = 0.0008, Fig. 3C). Time spent in the corners was also affected by treatment (F(1,48) = 4.831; *p* = 0.0328, *d* = 0.09) and an interaction (F(1,48) = 2.948; *p* = 0.0924, *d* = 0.05).

Time spent in the 20 cm center was reduced by HFD diet in VCD-treated females (*p* = 0.0329; Fig. 3D). Similarly, the number of entries made into the 10 cm center space was also altered by diet (F(1,47) = 5.992; *p* = 0.0182, *d* = 0.11) with HFD reducing 10 cm entries between VCD-treated mice (0.0136, Fig. 3E). The amount of time spent with either just the animals head or the torso center point having crossed the 10 cm center threshold exhibited trending diet effects (F(1,47) = 3.560; *p* = 0.0654, *d* = 0.06; F(1,45) = 3.089; *p* = 0.0856, *d* = 0.06) with VCD treatment also trending (*p* = 0.057). Additionally, there were also a treatment effects detected (F(1,48) = 4.834; p = 0.0328, *d* = 0.09) in percent entries in the 10 cm zone as VCD reduced percent entries between HFD diet groups (*p* = 0.0146; Fig. 3F). Our data suggests that in stressed female mice, VCD increases avoidance behaviors in a diet-dependent manner and that the combination of VCD and HFD amplified these effects, which may be due, in part, to an overall reduction in general locomotion. We have reported that a CVMS paradigm increases avoidance behaviors (reduces time spent in the center or increases time in the perimeter) in the OFT (Degroat et al., 2025).

**Fig. 4.**
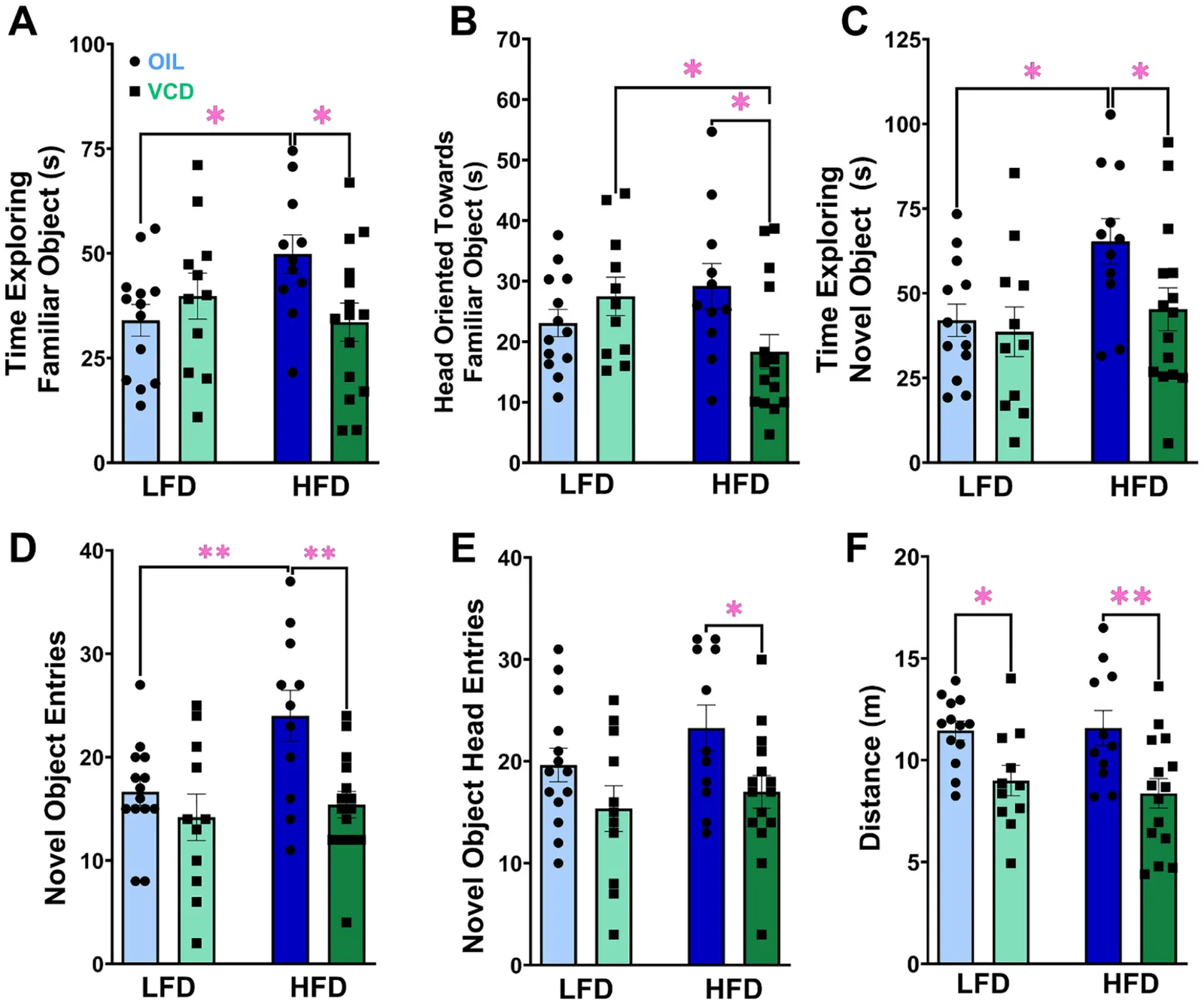
Novel object recognition. Cognitive and memory in oil- and VCD-treated females fed either LFD or HFD. (A) Total time (seconds) exploring familiar object (B) Total head entries into familiar object zone (C) Total time (seconds) exploring novel object (D) Total Novel object zone entries (E) Total head entries into novel object zone (F) Total distance (meters) traveled. VCD treatment impaired memory, with significant effects on distance traveled, speed, and novel object interactions. Data presented as mean +/−SEM and analyzed by two-way ANOVA with uncorrected Fisher’s LSD post-hoc comparisons (*, P = 0.01 to 0.05; **, P = 0.001 to 0.01).

Our next assay was the NOR to evaluate recognition memory (non-hippocampal, non-spatial). HFD increases the time spent with the familiar object in oil-treated females (*p* = 0.0222) but VCD reduced this time between HFD-fed groups (*p* = 0.0157; interaction: F(1,46) = 5.603; p = 0.0222, *d* = 0.10; Fig. 4A). VCD reduced the amount of time the animal’s heads was oriented towards or was directly exploring the familiar object between HFD-fed females (*p* = 0.0126) and HFD reduced the same parameter between VCD-treated females (*p* = 0.0337; interaction: F(1,46) = 6.446; p = 0.0146, *d* = 0.12; Fig. 4B). Similar to time spent with familiar object, the time spent in the novel object zone was affected by diet (F(1,46) = 5.576; *p* = 0.0225, *d* = 0.01) as time spent was elevated by HFD in oil-treated females (*p* = 0.0139) and reduced by VCD treatment in HFD-fed groups (*p* = 0.028; Fig. 4C). Entries into the novel object zone was higher in HFD-fed, oil-treated females compared to LFD-fed (*p* = 0.0059), which was reduced in VCD-treatment (*p* = 0.0013) as both diet (F(1,47) = 5.733; *p* = 0.0207, *d* = 0.09) and treatment (F(1,47) = 9.538; *p* = 0.0034, *d* = 0.15) influenced this parameter (Fig. 4D). Likewise, VCD-treatment in HFD-fed females reduced the number of head entries in the novel object zone (*p* = 0.024; treatment: F(1,47) = 7.587; *p* = 0.0083, *d* = 0.13; Fig. 4E). Similar effects (elevated in HFD-fed, oil-treated; reduced in HFD-fed, VCD-treated) were found in other parameters in the novel object (data not shown). Interestingly, distance traveled in the NOR was reduced by VCD treatment in both LFD- (*p* = 0.0197) and HFD-fed (*p* = 0.0021) females (F(1,46) = 16.02; *p* = 0.0002, *d* = 0.25; Fig. 4F). These data suggest that in stressed female mice, HFD alters hippocampal-independent memory but the depletion of follicles by VCD reduces these effects. In our previous VCD study with a HFD component (no stress), we found no effects of VCD on NOR parameters (Sui et al., 2023).

**Fig. 5.**
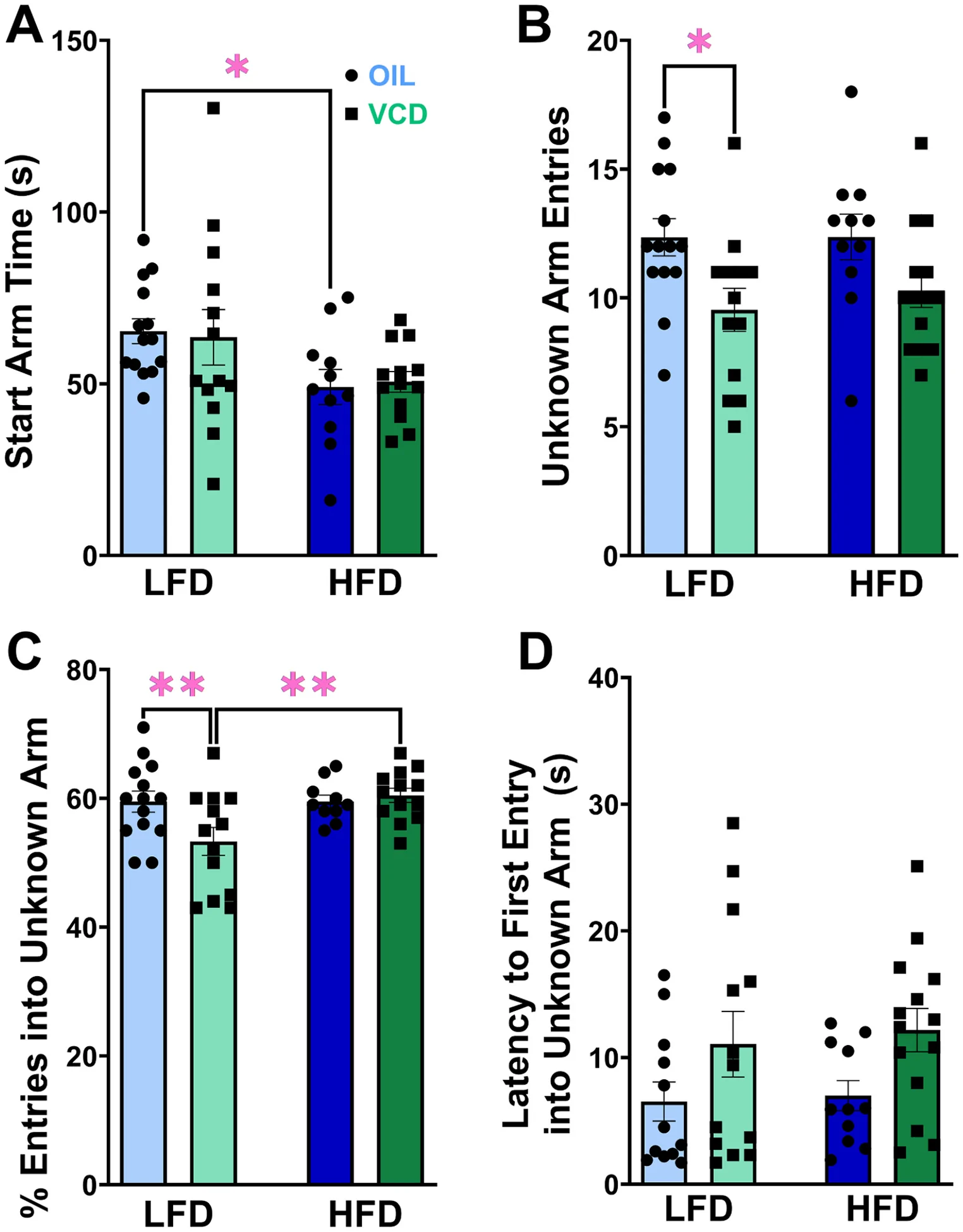
Y-maze. Spatial memory in oil- and VCD-treated females fed either LFD or HFD. All of the following figures were generated from the final 5-min exploration phase (A) Total amount of time (seconds) spent in Start arm (B) Total number of entries into Unknown arm (C) Percent entries into Unknown arm (D) Latency of time from start of test until first entry was made into unknown arm. VCD-treated mice showed impaired hippocampal-dependent memory, with significant diet and interaction effects on distance, speed, and unknown arm entries. Data presented as mean +/−SEM and analyzed by two-way ANOVA with uncorrected Fisher’s LSD post-hoc comparisons (*, P = 0.01 to 0.05; **, P = 0.001 to 0.01).

To exam hippocampal-dependent spatial memory, we used the Y-maze assay. Between oil-treated groups, HFD reduced the time spent in the start arm (*p* = 0.0385; diet: F(1,47) = 7.539; *p* = 0.0085, *d* = 0.14, Fig. 5A). For spatial memory, VCD-treated mice in LFD-fed females reduced unknown arm entries (*p* = 0.011) with a trend in HFD-fed females (*p* = 0.0686; treatment: F(1,48) = 10.07; *p* = 0.0026, *d* = 0.17; Fig. 5B). The percent entries into unknown arm was also reduced by VCD in LFD-fed females (*p* = 0.007) with a main effect of diet (F(1,46) = 4.851; *p* = 0.0327, *d* = 0.08) and an interaction effect (F(1,46) = 4.851; p = 0.0327, *d* = 0.08; Fig. 5C). Differences in LFD and VCD (p = 0.003) were also detected. While there were no pairwise differences, VCD increased the latency to first entry of the unknown arm (F(1,46) = 6.559; *p* = 0.0138, *d* = 0.12; Fig. 5D) with effects between both the LFD group (*p* = 0.09) and HFD (*p* = 0.064) groups treading towards significance. No difference was detected in center entries or time in the center (data not shown) suggesting there was no effect of indecision in the animal’s movements. These data suggest that overall, in stressed females, VCD suppressed hippocampal-dependent memory with greater effects in LFD-fed females. In our previous VCD study with a HFD component (no stress), we found a reduction in unknown arm entries in VCD-treated females compared to vehicle-treated indicating deficits in spatial memory (Sui et al., 2023).

**Fig. 6.**
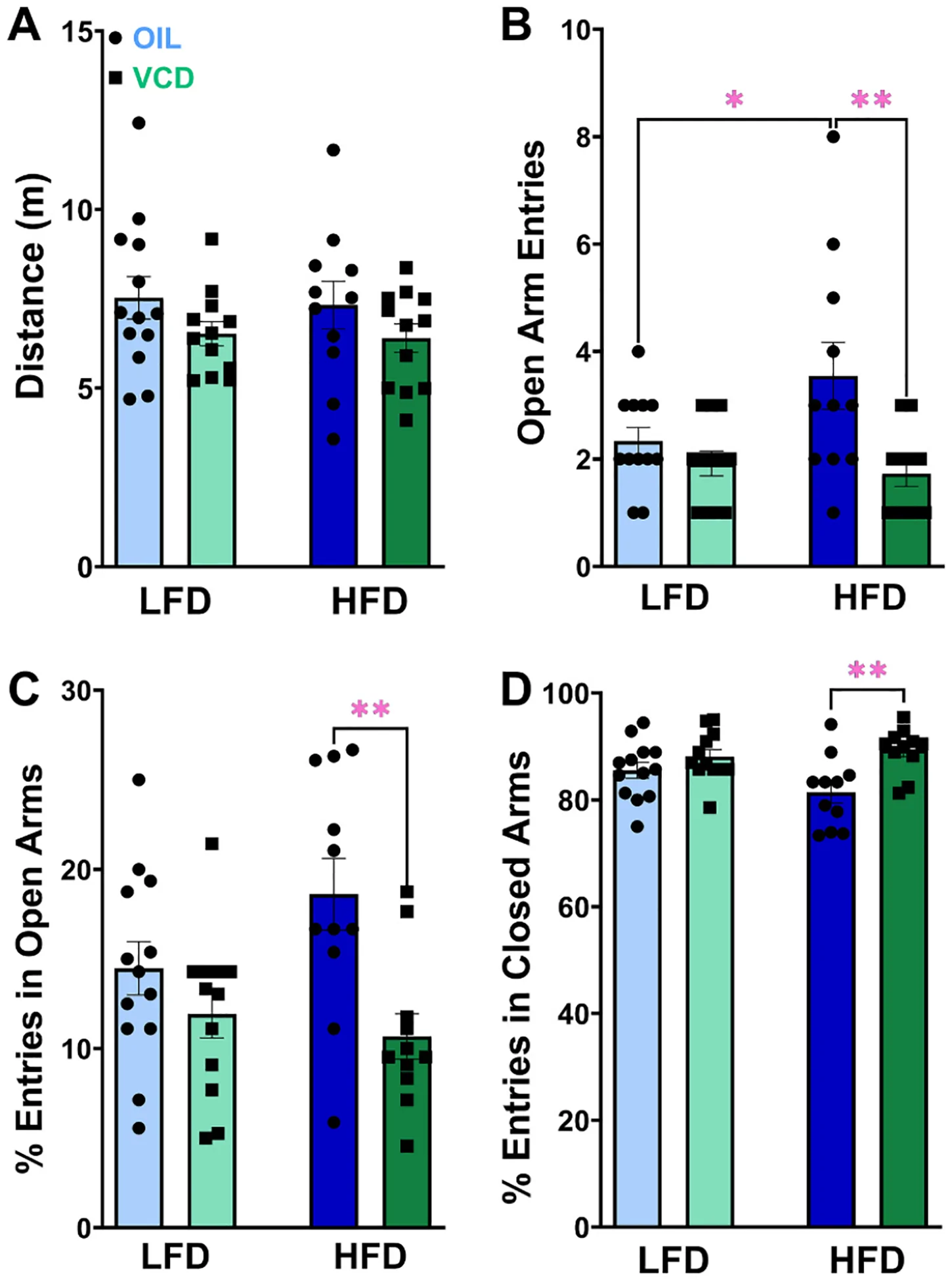
Elevated Plus maze. Avoidance behavior in oil- and VCD-treated females fed either LFD or HFD. (A) Total distance traveled (meters) throughout test (B) Total amount of open arm entries (C) Percent entries into open arms (D) Percent entries into closed arms. HFD-fed VCD-treated mice showed anxiety-like behavior, spending less time in the open arm. Data presented as mean +/−SEM and analyzed by two-way ANOVA with uncorrected Fisher’s LSD post-hoc comparisons (*, P = 0.01 to 0.05; **, P = 0.001 to 0.01).

We used the EPM to continue our investigation into avoidance behaviors. We found no differences in time spent in the closed arms or number of entries into closed arms or in distance traveled (Fig. 6A). However, we did find a treatment effect (F(1,44) = 4.362; *p* = 0.0426, *d* = 0.09) in the distance traveled in the closed arm (data not shown). Similar to other assays, HFD increased entries into open arms in oil-treated females (*p* = 0.0239), which was reduced by VCD treatment (*p* = 0.0013; treatment: (F(1,42) = 9.334; *p* = 0.0039, *d* = 0.16; Fig. 6B). VCD also reduced percent entries into the open arms in HFD-fed females (p = 0.001; treatment: F(1,43) = 11.56; *p* = 0.0015, *d* = 0.2; Fig. 6C). Additionally, there was a trending effect between oil-treated females (*p* = 0.0626). VCD treatment also modestly increased the percent entries into the closed arms in HFD-fed females (p = 0.001; F(1,43) = 11.56; p = 0.0015, *d* = 0.20; Fig. 6D). Similar to the previous assays, VCD treatment in HFD-fed stressed females increased avoidance behavior. We have also reported that a CVMS paradigm increases avoidance behaviors (increases time spent in the closed arms) in the EPM (Degroat et al., 2025).

During NSF testing, only three out of 14 animals in the LFD-fed, oil-treated group consumed any food within the novel arena with a latency of 442.5 ± 32.8 s. In the home cage, 9 of the 14 females in the LFD-oil group consumed food with a latency of 130.0 ± 23.6 s, 8 of the 11 females in HFD-oil group consumed food with a latency of 125.6 ± 30.9 s. Amongst VCD-treated females, 4 of 10 LFD-fed females ate the pellet in the home age with a latency of 89.6 ± 30.9 s and 11 of 16 HFD-fed females consumed the pellet with a latency of 109.8 ± 21.4 s. There were no significant differences between groups in the latency to eat in the home cage. In our previous CVMS study, stress did not alter avoidance behaviors (latency to eat in novel arena) in either proestrous/estrous or diestrous females in the NSF (Degroat et al., 2025).

### 3.3. Uterine weights, CORT, and metabolic hormones

**Fig. 7.**
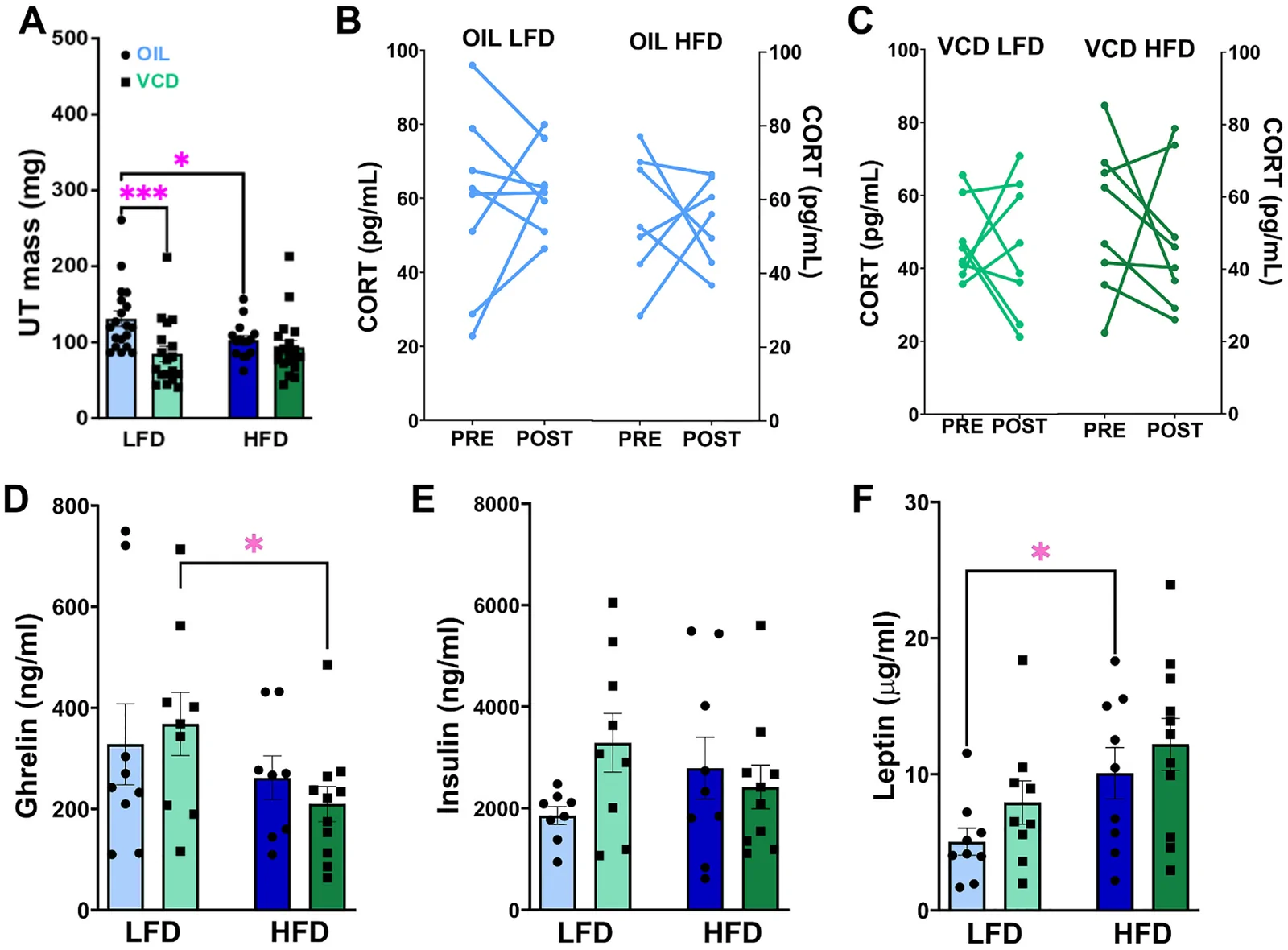
Metabolic and stress hormone assay data. Metabolic and stress hormones in oil- and VCD-treated females fed either LFD or HFD. (A) Uterine mass (mg) at sac (B) Corticosterone level comparison of pre and post CVMS exposure in OIL/LFD and OIL/HFD (C) Corticosterone level comparison of pre and post CVMS exposure in VCD/LFD and VCD/HFD (D) Detected plasma Ghrelin levels (ng/ml) (E) Insulin (ng/ml) and (F) Leptin (ug/ml) at decapitation. Data presented as mean +/−SEM and analyzed by two-way ANOVA uncorrected Fisher’s LSD post-hoc comparisons (*, P = 0.01 to 0.05; ***, P = 0.0001 to 0.001).

Uterine weights collected at euthanasia were significantly lower in VCD-treated females fed LFD-diet (*p* = 0.0006) and reduced in HFD feed oil-treated females (*p* = 0.0431; treatment: F(1,67) = 8.945; p = 0.0039, *d* = 0.11; Fig. 7A) when compared to LFD fed oil-treated counterparts. Prior to stress, CORT levels were similar across all groups (data not shown). Post-stress CORT levels were impacted by VCD treatment (F(1, 27) = 4.499, *p* = 0.0432, *d* = 0.14), as VCD decreases CORT in LFD-fed females (*p* = 0.0350), indicating that the depletion of follicles impacts corticosterone production (Fig. 7B & C). Ghrelin levels were trending towards having a diet effect (F(1,33) = 3.881, *p* = 0.0573, *d* = 0.1) with a modest decrease in HFD-fed VCD treated females compared to LFD-fed (*p* = 0.0491; Fig. 7D). There were no main treatment effects in insulin levels but a trending interaction effect (F(1,32) = 3.386, *p* = 0.0750, *d* = 0.09; Fig. 7E). Leptin levels were elevated in HFD-fed mice overall (F (1,34) = 7.630, *p* = 0.0092, *d* = 0.17, Fig. 7F) with HFD-fed, oil-treated females expressing higher leptin levels compared to LFD-fed (*p* = 0.0468) while the effect of HFD in VCD-treated females was trending (*p* = 0.0747).

### 3.4. adBNST transcriptome

**Fig. 8.**
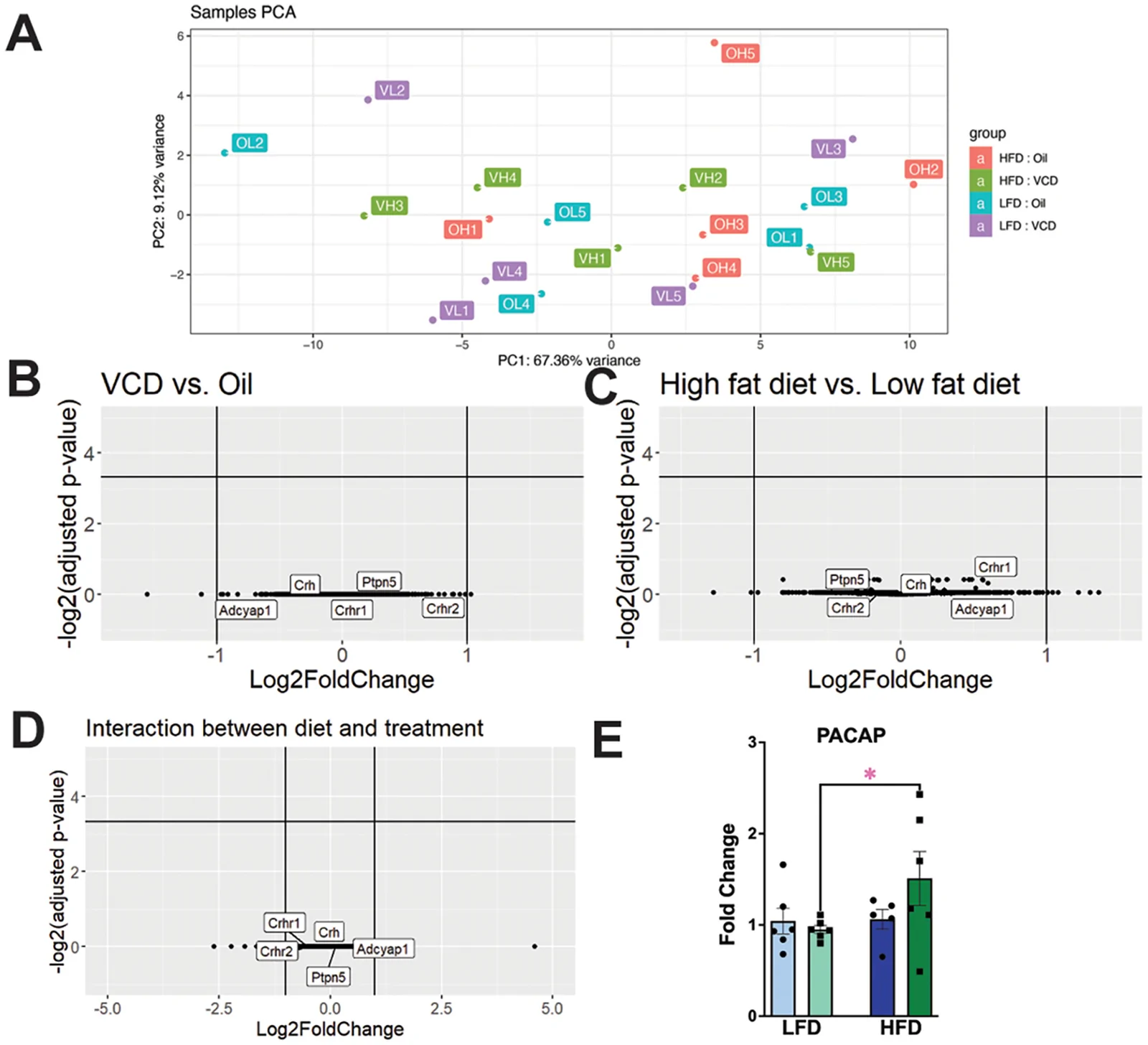
Gene expression. RNA-Sequencing and qPCR of the adBNST in oil- and VCD-treated females fed either LFD or HFD. (A) PCA plot of samples. Volcano plots for comparisons between VCD treatments (B), between diets (C) and the interaction (D). (E) PACAP (*Adcyap1)* gene expression in adBNST. Data presented as mean +/−SEM and analyzed by two-way ANOVA with uncorrected Fisher’s LSD post-hoc comparisons (*, P = 0.01 to 0.05).

Analysis of the adBNST transcriptome suggested that neither diet nor depletion of follicles (VCD) differentially alters the gene expression in stressed females. The PCA plot (Fig. 8A) and volcano plots (Fig. 8B–D) revealed no significant differentially expressed genes (DEGs) in the transcriptome. These findings indicate that the lack of ovarian hormones and/or DIO do not greatly influence the transcriptional response in this region to chronic stressors. We have recently reported that CVMS does not influence the transcriptome (only one gene) in 14-week-old, cycling females (Degroat et al., 2025). However, when using a targeted approach to study CRH-associated gene expression, we found that the expression of *Adcyap1* (pituitary adenylate cyclase-activating polypeptide (PACAP), a CRH activator, was increased by HFD in VCD-treated mice (*p* = 0.0357; Fig. 8C). Lastly, we did not see any effects in any other genes we had screened for gene expression (*Crh, Crhr1, Crhr2, Ptpn5* (STEP) or *Pacap1r*).

## 4. Discussion

In this study, we explored the interactions between DIO and menopause, in a mouse model of advance ovarian failure by VCD treatment, and their effects on metabolic, behavioral, and transcriptome responses after 6 weeks of chronic stress. We aimed to gain further understanding on how these factors interact to influence avoidance behavior, cognition, and metabolism, as well as gene expression in the adBNST, a key brain region involved in stress responses. Our results indicate the detrimental impact of HFD and VCD on body composition, avoidance behavior, memory impairment, and metabolic hormone levels in mice. However, the transcriptome of the adBNST was not affected. Herein we discuss the metabolic outcomes, followed by behavioral findings, hormonal responses, and the transcriptomic data.

The perimenopause transition and menopause both induce metabolic changes that are associated with increased risk for mood disorders and cognitive decline. In cisgender women, menopause is associated with increased central adiposity and metabolic syndrome, increasing the likelihood of obesity, largely because hormonal shifts impact energy balance and fat distribution, (Premaor et al., 2010; Mauvais-Jarvis et al., 2013; Ford et al., 2017), paralling our findings in mice. Yet, a diet low in fat may increase the risk of weight gain during menopause (Ford et al., 2017). Our study of the VCD rodent model of menopause found changes in metabolism including increased weight gain, fat mass, and feeding efficiency in HFD-fed, VCD-treated mice, aligning with previous studies linking DIO and menopause to metabolic dysregulation in rodents (Patterson et al., 2013; Kumari et al., 2024). VCD treatment alone induces weight gain and glucose intolerance, which is amplified by HFD, leading to disruption of blood biomarkers of metabolic health and hypothalamic expression of genes involved in metabolism (Abi-Ghanem et al., 2024). Stress exposure in menopausal women also increases the risk for cardiometabolic diseases (hypertension, metabolic syndrome). In rats, VCD when coupled with a 10-day chronic unpredictable stress paradigm negatively impacted cardiovascular outcomes such as an increase in the overall spontaneous baroreflex sensitivity (Lorenzon et al., 2019). In our previous VCD & HFD study, we reported elevated blood pressure and heart rates in aged, VCD-treated females (Sui et al., 2023). This highlights the importance of diet in managing the postmenopausal risk for obesity and the impact of the hormonal changes due to menopause.

In mice, increased central leptin impairs leptin signaling and promotes further weight gain influencing both insulin and leptin regulation (Zhang and Scarpace, 2006). This is also seen when exogenous leptin administration in weight-reduced individuals restores normal energy expenditure and sympathetic nervous system function (Rosenbaum et al., 2005). Leptin and insulin, both elevated in obesity, are known to influence cognitive functions and anxiety (Rosenbaum et al., 2005). In studies examining DIO and aging, elevated leptin levels contribute to leptin resistance, which exacerbates obesity (Zhang and Scarpace, 2006). While leptin is primarily associated with energy homeostasis, it also has roles in memory and avoidance behaviors, suggesting that the elevated levels seen in our HFD mice could contribute to the observed cognitive impairments and anxiety-like (avoidance) behavior (Witte et al., 2016). Our study further confirms findings that VCD-induced metabolic disruptions are exacerbated by HFD feeding in mice as previously reported (Sui et al., 2023; Kumari et al., 2024; Abi-Ghanem et al., 2024).

Previous studies on menopause models have reported increased anxiety-like behaviors and impaired avoidance learning, consistent with our findings in the OFT and EPM (Reis et al., 2014; Karisetty et al., 2017; Kang et al., 2023). Administration of estrogen attenuated these behaviors, indicating that hormonal changes significantly impact stress-related avoidance and can be mitigated by estrogen replacement (Karisetty et al., 2017; Kuh et al., 2018). Our data, showing heightened anxiety in HFD-fed, VCD-treated mice, aligns with these reports and underscores the interaction between metabolic state and menopause. Postmenopausal people are at greater risk for anxiety disorders, particularly when obesity is present, reflecting similar trends seen in our animal model (Bromberger et al., 2013; Karisetty et al., 2017; Tomiyama et al., 2018; Kuh et al., 2018). Other research has identified anxiety-like (avoidance) behaviors in the VCD mouse model, further finding that treadmill exercise reduces these anxiety-life behaviors and neuronal apoptosis, with the improvements positively correlated with increased levels of circulating osteocalcin (Kang et al., 2023). In our published studies using the same CVMS paradigm, we consistently found that, in terms of avoidance behavior, CVMS augmented avoidance behaviors (OFT, EPM, NSF) in young intact females in vaginal proestrus or estrus but not diestrous females (Degroat et al., 2025). One explanation for the stage-dependent effects is that unstressed diestrous females were already more avoidant than unstressed proestrous/estrous females, thus exhibiting a floor-effect on avoidance and locomotion. Sensitivity to our CVMS paradigm appears to be hormone-mediated in females, is heightened in mice with no gonadal steroid production (menopause) or high steroid flux states (proestrus/estrus), and attenuated in low steroid states (diestrus).

Cognitive decline is often reported during the menopausal transition, and obesity is a known risk factor for accelerated cognitive aging (Anacker and Hen, 2017). Female rodents are more resilient to chronic stressors than males in terms of spatial memory and object recognition and in some reports, stress may augment memory in female rodents (Luine et al., 2017). In a study in aged (20+ months) female mice, chronic stress resulted in impaired memory (Lotan et al., 2018). In a model of Alzheimer’s (5xFAD AD mice), chronic stress augmented spatial memory impairments in the 5xFAD female mice (Shlomi-Loubaton et al., 2025). Our results from the Y-Maze and NOR tests indicate that VCD treatment impairs memory, with a more pronounced detrimental impact in HFD-fed mice. These findings are consistent with other studies demonstrating that menopause exacerbates cognitive decline, particularly in memory tasks reliant on hippocampal function (Elsabagh et al., 2007; Bromberger et al., 2013; Premaor et al., 2010; Mauvais-Jarvis et al., 2013; Ford et al., 2017; Kuh et al., 2018; Kang et al., 2023). Our study using the VCD model and HFD feeding found that a HFD low in linoleic acid, a common polyunsaturated fatty acid in Western diets, reduced spatial memory in VCD-treated, middle-aged female mice compared to vehicle-treated females (Sui et al., 2023). Intact female mice exhibit improved cognitive and emotional resilience compared to ovariectomized mice with more pronounced cognitive deficits and depressive behaviors in response to stress (Karisetty et al., 2017). In VCD-treated rats, memory was enhanced by estradiol during learning (Koebele et al., 2020). In humans, studies have found that while attention, verbal fluency, and memory remain stable, executive function significantly declines in women during late postmenopause, potentially due to hormonal changes (Elsabagh et al., 2007). Another clinical study found that estradiol positively influenced cognitive functions such as verbal memory, working memory, and selective attention in healthy postmenopausal women, but these benefits were diminished when cortisol levels were elevated due to stress (Baker et al., 2012). Additionally, estradiol alone had a favorable effect on amyloid-β biomarker associated with neurodegenerative diseases, but this effect was counteracted when cortisol was also elevated overall suggesting that elevated stress hormone levels can reduce the cognitive and physiological benefits of estradiol in postmenopausal women (Baker et al., 2012). Alternatively, previous studies have tested conjugated equine estrogens in rats, finding that while the treatment improved cognition in surgically menopausal rats, it did not benefit cognition in transitionally menopausal rats, which more closely mimics the experience of cisgender women (Acosta et al., 2010). Again, we acknowledge that variations in our findings compared to other studies could be due to the specific age at the beginning of treatment, differences in the timing and type of diet intervention, species, or the stress paradigm.

High fat diets alone can impair cognitive function, avoidance behavior and alter feeding behavior in both humans and animal models (Spencer et al., 2017; Parande et al., 2022; Sui et al., 2023; Kang et al., 2023). In an AD mouse model treated with VCD and fed control or HFD, the combination of HFD and VCD treatment led to impaired recognition memory potentially by augmenting hippocampal amyloid pathology (Abi-Ghanem et al., 2024), while some studies find that HFD impaired fear memory extinction in male mice but not in female mice (Shetty et al., 2024). Excessive consumption of high-sugar diets impairs cognitive function and memory, particularly affecting the hippocampus, which is crucial for spatial and episodic memory (Reichelt et al., 2018). Diet induced obesity is widely recognized to negatively impact cognitive function and promote anxiety-like behavior, with mechanisms involving both peripheral and central insulin resistance and altered leptin signaling (Spencer et al., 2017; Tomiyama et al., 2018; Parande et al., 2022). Early-life overfeeding and high-fat diets can exacerbate neuroinflammation, impair memory, and increase susceptibility to depressive-like behaviors (Spencer et al., 2017). Indeed, adolescent mice (4–8 weeks) exposed to chronic stress and HFD exhibit greater avoidance and impaired spatial memory (Lippi, 2021). However, chronic stress and DIO do not consistently impact cognition in female rodents, especially when compared to males (Prochnik et al., 2022). In the context of menopause, these effects may be magnified, as seen in our VCD-treated, HFD-fed mice. The literature suggests that DIO can exacerbate menopause-related cognitive and emotional disturbances, which aligns with our observations (van Dijk et al., 2015; Reichelt et al., 2018; Sui et al., 2023). A previous study found that changes in gut microbiota and short-chain fatty acids linked to estrogen levels were associated with cognitive impairments and weight changes, highlighting the connection between diet, menopause, and cognitive function (Zeibich et al., 2021). Our data may differ from other studies due to specific diet composition, duration of diet treatment, and the duration and type of chronic stress model.

Our RNA-sequencing analysis of the adBNST revealed no significant DEGs, which agrees with previous studies in stress models where chronic stress does not alter transcriptome in this region in female mice (Degroat et al., 2024, 2025). This lack of effect may be due to the specific time point we selected for analysis or the possibility that the adBNST’s transcriptional response requires more sensitive detection methods. It may also be due to the fact that because this region is sensitive to stress in rodents, all the mice being stressed took precedence over any potential differences due to diet or VCD treatment. However, in our recent study using a similar tissue collection time in young cycling intact females exposed to CVMS, we found that only one gene (*Rbm3*) was differentially regulated in the female adBNST unlike in intact males where 217 genes were differentially expressed (Degroat et al., 2025). Collectively, our two studies demonstrate that the transcriptome of the adBNST in females is not altered by stress, hormone state, or diet. Furthermore, our lab has reported that CVMS enhances CRH neuronal excitability in the adBNST via protein kinase A (PKA)-dependent CRHR1 signaling in males (Hu et al., 2020; Maita et al., 2022; Degroat et al., 2025) but not in females (Degroat et al., 2025), highlighting the need to study the stress response across the sexes and in all steroidal states.

PACAP interacts with CRH signaling in the BNST, with changes in PACAP and CRHR1 expression contributing to stress-induced anxiety-like behaviors (Stroth et al., 2011; Hu et al., 2020; Lepeak et al., 2023). Our previous study in intact males found that PACAP was upregulated in the adBNST of male mice by CVMS, both in terms of mRNA and protein (Hu et al., 2020). In the current study, PACAP expression was increased only HFD-fed, VCD-treated mice suggesting that diet may only influence CRH-associated signaling in stressful conditions. A study in female rats found that PACAP infusion into the BNST increases plasma corticosterone levels. The effect was specific to the BNST and was not blocked by CRH receptor antagonism, suggesting PACAP’s key role in stress response regulation (Lezak et al., 2014). Further studies by this group found that a 7-day chronic variable stress paradigm did not enhance the startle response in female rats after infusion of PACAP into the BNST as was found in male rats (King et al., 2017). These data support our findings that stress alone does not alter PACAP in females but stress plus HFD plus estrogen deficiency may.

A limitation of our study is the absence of unstressed control groups, which would have allowed for further distinction between the effects of diet, menopause, and chronic stress. However, even without the no-stress control group, we can compare how diet and a “menopausal state” in a mouse influence the behavioral response to chronic stress. Future studies should also consider the inclusion of female mice at different stages of the menopausal transition (perimenopausal) and their response to chronic stress and DIO to better model the variability seen in clinical studies and provide context for studies on strategies to manage symptoms of menopause. Other studies should examine different stress paradigms, such as acute variable stress models for post-traumatic syndrome disorder or chronic social instability stress, and DIO prior to VCD administration. Other limitations include the relevance of a chemically-induced mouse model of menopause to the naturally occurring depletion of follicles and cessation of menstrual cycles in cisgender women. While the VCD model does mimic the major aspects of the perimenopausal transition and a similar endocrine profile, mice do not experience such a state naturally nor do they experience DIO. Thus, their physiological and behavioral responses to these influences may not fully recapitulate the human condition.

## 5. Summary

Our study, suggests that under stressful conditions, diet and/or metabolic condition influence the cognitive and avoidance (anxiety-like) behavioral responses in “menopausal” mice. We first confirmed the disruption to metabolism prior to stress due to both the VCD (menopause) treatment and due to HFD feeding with greater adiposity, elevated glucose and leptin, and slower metabolism (feeding efficiency). Behavioral outcomes revealed augmented avoidance behavior and impaired cognition when HFD feeding is coupled with VCD treatment, implicating the cognitive and mood repercussions of estrogen deficiency and DIO. These findings also highlight the potential resilience that a HFD provides against some estrogen deficiency-related cognitive impairments such as spatial memory. The relevance of our study to women’s health is limited by the differences between the menstrual cycle in humans and the estrous cycle of mice, which do not experience menopause but rather reproductive senescence as they age. The VCD model does recapitulate one aspect of menopause, the depletion of follicles, but it does not fully emulate menopause. Thus, our conclusions should be interpreted carefully within the scope of the study design. Regardless, these findings help provide context to the interactions between diet, menopause, and stress suggesting that postmenopausal women with obesity may be at heightened risk for anxiety and cognitive decline, especially if they experience high levels of stress. Future studies should consider the interactions of these factors when exploring therapeutic strategies to mitigate these risks in menopausal people.

## Data Availability

Data will be made available on request.
